# Diffusion–Perfusion Mismatch: An Opportunity for Improvement in Cortical Function

**DOI:** 10.3389/fneur.2014.00280

**Published:** 2015-01-14

**Authors:** Melissa Motta, Amanda Ramadan, Argye E. Hillis, Rebecca F. Gottesman, Richard Leigh

**Affiliations:** ^1^R Adams Shock Trauma Center, University of Maryland School of Medicine, Baltimore, MD, USA; ^2^Johns Hopkins University School of Medicine, Baltimore, MD, USA; ^3^Department of Neurology, Johns Hopkins University School of Medicine, Baltimore, MD, USA

**Keywords:** diffusion–perfusion mismatch, acute ischemic stroke, penumbra, NIHSS, functional outcome

## Abstract

**Objective:** There has been controversy over whether diffusion–perfusion mismatch provides a biomarker for the ischemic penumbra. In the context of clinical stroke trials, regions of the diffusion–perfusion mismatch that do not progress to infarct in the absence of reperfusion are considered to represent “benign oligemia.” However, at least in some cases (particularly large vessel stenosis), some of this hypoperfused tissue may remain dysfunctional for a prolonged period without progressing to infarct and may recover function if eventually reperfused. We hypothesized that patients with persistent diffusion–perfusion mismatch using a hypoperfusion threshold of 4–5.9 s delay on time-to-peak (TTP) maps at least sometimes have persistent cognitive deficits relative to those who show some reperfusion of this hypoperfused tissue.

**Methods:** We tested this hypothesis in 38 patients with acute ischemic stroke who had simple cognitive tests (naming or line cancelation) and MRI with diffusion and perfusion imaging within 24 h of onset and again within 10 days, most of whom had large vessel stenosis or occlusion.

**Results:** A persistent perfusion deficit of 4–5.9 s delay in TTP on follow up MRI was associated with a persistent cognitive deficit at that time point (*p* < 0.001). When we evaluated only patients who did not have infarct growth (*n* = 14), persistent hypoperfusion (persistent mismatch) was associated with a lack of cognitive improvement compared with those who had reperfused. The initial volume of hypoperfusion did not correlate with the later infarct volume (progression to infarct), but change in volume of hypoperfusion correlated with change in cognitive performance (*p* = 0.0001). Moreover, multivariable regression showed that the change in volume of hypoperfused tissue of 4–5.9 s delay (*p* = 0.002), and change in volume of ischemic tissue on diffusion weighted imaging (*p* = 0.02) were independently associated with change in cognitive function.

**Conclusion:** Our results provide additional evidence that non-infarcted tissue with a TTP delay of 4–5.9 s may be associated with persistent deficits, even if it does not always result in imminent progression to infarct. This tissue may represent the occasional opportunity to intervene to improve function even days after onset of symptoms.

## Introduction

At the onset of an ischemic stroke, an occluded blood vessel results in diminished blood flow to a region of brain tissue, resulting in cerebral ischemia. Ischemic tissue becomes electrically dysfunctional and can progress to infarction. The term ischemic penumbra was coined by Astrup and colleagues in the 1970s to characterize a state of brain tissue in which “neurons remain structurally intact but functionally inactive” and in which a regional increase in cerebral blood flow can restore this activity (1). From experiments in baboons, they reported dual thresholds for ischemia; the threshold for cell death (signaled by the release of K+) was markedly lower than the threshold for complete electrical failure of neurons. In its original use, the “penumbra” differentiated the outer rim, or “half shadow” of tissue, which had reached the threshold for electrical failure, from the inner core of infarction, which had reached the threshold for sustained energy failure resulting in ion pump failure (2). Thus, acute occlusion of a large cerebral artery typically results in region of diminished blood flow in which the central core experiences the most severe ischemia and can rapidly progress to infarct. Surrounding the core there may be a region of hypoperfusion that is ischemic but not infarcted; this area is commonly referred to as the ischemic penumbra. The penumbra is electrically dysfunctional tissue that, when reperfused, will regain function.

With the introduction of positron emission tomography (PET) imaging, regional cerebral blood flow could be measured in humans, and the thresholds for ischemia and dysfunction were further defined. The concept of ischemic penumbra was elaborated by Muir and colleagues (3) to include the following criteria: “(a) hypoperfusion <20 mL/100 g/min; (b) abnormal neuronal function documented by a correlation with acute clinical deficit; (c) physiological and/or biochemical characteristics consistent with cellular dysfunction but not death; (d) uncertain fate; and (e) salvage of this tissue is correlated with better clinical recovery” (p. 761). Surrounding the layer of ischemic penumbra (reduced cerebral blood flow to <20 mL/100 g/min) exhibiting impaired neural function but preserved tissue integrity, it was recognized there was often a third zone of reduced blood flow. This zone was defined as tissue with relatively small reductions in blood flow of 20–50 mL/100 g/min. It was assumed this tissue would maintain function for a protracted time and would be unlikely to proceed to infarction, and was thus labeled “benign oligemia.”

With the advent of multimodal MRI, a different biomarker for the penumbra was proposed (3, 4). Diffusion weighted imaging (DWI), which measures the movement of water in brain tissue, was used to delineate ischemic tissue that had sustained energy failure and thus exhibited diffusion restriction. Perfusion weighted imaging (PWI), which uses a bolus tracking dynamic scan to measure the delivery of blood to the brain, was used to delineate the region of ischemia. Thus, the difference between the DWI lesion and the PWI lesion was postulated to be a biomarker for the ischemic penumbra. This biomarker has held up when compared to biomarkers of penumbra obtained from PET imaging (5–7).

As the field of acute stroke treatment was emerging, it was postulated that the diffusion–perfusion mismatch would guide treatment and predict response to therapy. In order to use this biomarker in such a manner it had to be adapted into a predictive model. Initially, it was hypothesized that in the absence of acute vessel recanalization, the infarcted core would grow into the ischemic penumbra and the opportunity for clinical recovery would be lost. This hypothesis has been the driving force behind most MRI-guided acute stroke research.

In the context of the MRI-guided therapies, the term “penumbra” has now come to be understood as “tissue at risk.” A number of authors have pointed out that the diffusion–perfusion mismatch includes both hypoperfused tissue that will imminently infarct (“tissue at risk”) and hypoperfused tissue that will survive despite hypoperfusion (“benign oligemia”) (8). In the context of MRI-guided therapies, benign oligemia is thought to be irrelevant, and it is excluded from the lesion targeted for reperfusion therapy.

It has been shown that MRI based thresholds of hypoperfusion are capable of separating benign oligemia from tissue that is at-risk of imminent infarction. The threshold for distinguishing benign oligemia from tissue at risk has varied across studies and changed over time; however, the most frequently used threshold in the current literature is a time-to-maximum of 6 s (9). However, there might be tissue in between tissue at risk for imminent progression to infarct and benign oligemia – tissue that will remain dysfunctional if blood flow remains compromised and will regain function if blood flow is restored. A number of case reports of improvement in function after reperfusion by carotid endarterectomy, carotid stenting, or blood pressure elevation even days after stroke onset indicate the existence of this tissue that exists in a state of limbo between adequate blood flow for function and immediate risk of infarction (10–12). It is not entirely clear how this dysfunctional tissue survives over days with “misery perfusion”; it may do so by collateral blood flow or intermittent blood flow from the main arterial supply. Nevertheless, MRI might be able to define the thresholds of this hypoperfused, dysfunctional tissue. Data from the case reports of reversible function days after stroke indicate that a delay in time-to-peak (TTP) arrival of contrast of 4–5.9 s might represent tissue that is dysfunctional but not always at immediate risk of progression to infarction (particularly in cases of large vessel stenosis). This segment of penumbral tissue is important to define, as it may represent an opportunity to improve at least cortical function in special cases long after the typical window for reperfusion therapies.

A study using PET as the gold standard found that a TTP threshold of 4.2 s (or Tmax threshold of 5.2 s) corresponds to penumbra as operationally defined above (see definition by Muir and colleagues), and indicated that the TTP threshold might be a more stable (3, 5). Another study found that non-deconvolved TTP outperformed Tmax in distinguishing oligemia from tissue at risk (13), and using local arterial input functions did not improve the predictive performance of the algorithms over conventional methods. Other studies have shown that reperfusion of penumbral tissue identified on MRI results in not only tissue salvage but also improved outcomes (14). However, the clinical relevance of a persistent hypoperfusion in the range of TTP of 4–5.9 s delay has not been adequately addressed.

As noted, a few studies have shown that individuals with persistent hypoperfused tissue of 4–5.9 s delay in TTP have shown improved function with reperfusion (10–12). Thus, these studies illustrate that the diffusion–perfusion mismatch defined by MRI can reveal penumbra as originally described by Astrup and colleagues in primates – marginally perfused, dysfunctional tissue that can regain function if reperfused (3). These results mirror results of other studies showing improvement in function with reperfusion of penumbra defined by PET or CT perfusion (15–19). What these previous studies did not do, however, was show that persistent diffusion–perfusion mismatch, defined by hypoperfusion thresholds of 4–5.9 s delay in TTP, was associated with persistent dysfunction. Tissue within this range of TTP is often considered to be within the “benign oligemia” range, because it does not always progress to infarct. Persistent hypoperfused tissue of 4–5.9 s delay in TTP beyond the infarct that is associated with persistent deficits, in the absence of infarct growth, would provide evidence that hypoperfused tissue of 4–5.9 s delay in TTP may not be so benign.

Here, we address the question, “Is benign oligemia really benign?” We hypothesized that patients with a persistent diffusion–perfusion mismatch defined using upper and lower hypoperfusion thresholds of TTP 4–5.9 s delay have persistent dysfunctional tissue and poorer performance on cognitive tests when compared with patients who have reperfused, whether or not there is growth of the infarct. We studied this hypothesis by evaluating changes in volumes of infarct and hypoperfusion in a cohort of patients who underwent serial imaging and cognitive testing over several days during hospitalization for an acute ischemic stroke. Most of these patients had large vessel stenosis or occlusion, which carries the greatest risk for persistence of a diffusion–perfusion mismatch.

## Materials and Methods

Subjects were identified from a database of acute ischemic stroke patients who had been recruited from the inpatient stroke service under an IRB approved protocol to undergo serial cognitive and MRI testing, utilizing the following criteria: (1) clinical diagnosis of unilateral anterior circulation stroke, (2) cognitive testing and MRI scan performed within 24 h at two time points within 10 days, and (3) adequate quality DWI and PWI images for volumetric analysis at both time points. Patients had a variety of acute stroke interventions, often attempts to restore perfusion, including induced blood pressure elevation, carotid stenting, urgent endarterectomy, and intraarterial thrombolysis, at the discretion of the primary clinical team.

Cognitive testing consisted of a picture naming task for left hemisphere strokes and a line cancelation test for right hemisphere strokes both of which were scored as a percent error. The line cancelation test usually requires <2 min to administer. The picture naming test usually requires <10 min to administer. These tests are described in previous papers (14). For both, 10% error rate represents >3 SD from mean for normal controls (14).

Volumes of ischemia on DWI were calculated by manual delineation of a region of interest (ROI) that was bright on DWI and dark on apparent diffusion coefficient (ADC) maps. Volumes of hypoperfusion on PWI were calculated by manually segmenting thresholded TTP maps. We calculated volumes of tissue with delay in TTP of 4–5.9 and ≥6 s delay relative to normal tissue (the homologous voxels in the contralateral hemisphere). Perfusion source images were exported as a DICOM time series using OsiriX software. Then, TTP maps were generated and analyzed by ImageJ software^1^ by a technician blinded to cognitive test scores. TTP maps were calculated in seconds beyond normal using the homologous region in the unaffected hemisphere as a reference. TTP maps were used instead of Tmax maps as they have been shown to be as effective in detecting perfusion abnormalities and are simple and reproducible (5). We chose two separate thresholds to determine the volume of tissue with 4–5.9 s delay in TTP (tissue we hypothesized to be potentially viable, but dysfunctional), and tissue with ≥6 s delay in TTP (tissue more likely to be at immediately risk to progress to infarct).

Lack of reperfusion was defined as a <10% reduction in the PWI lesion volume. Infarct growth was defined as a >10% increase in DWI lesion volume. We chose these small thresholds of volume change in order to evaluate the effects of failure to reperfuse, in the absence of infarct growth (i.e., the persistence of diffusion–perfusion mismatch, using thresholds of hypoperfusion of TTP delay 4–5.9 or ≥6 s). Some treatment studies define “reperfusion” as >50% (or greater) reduction in PWI volume (14, 20). However, in our study we did not want patients with partial reperfusion, who may also have partial improvement, to contaminate the effect of sustained diffusion–perfusion mismatch, which is the target population for this study. Therefore, relatively strict definitions for lack of reperfusion and infarct growth were used.

**Figure 1:**
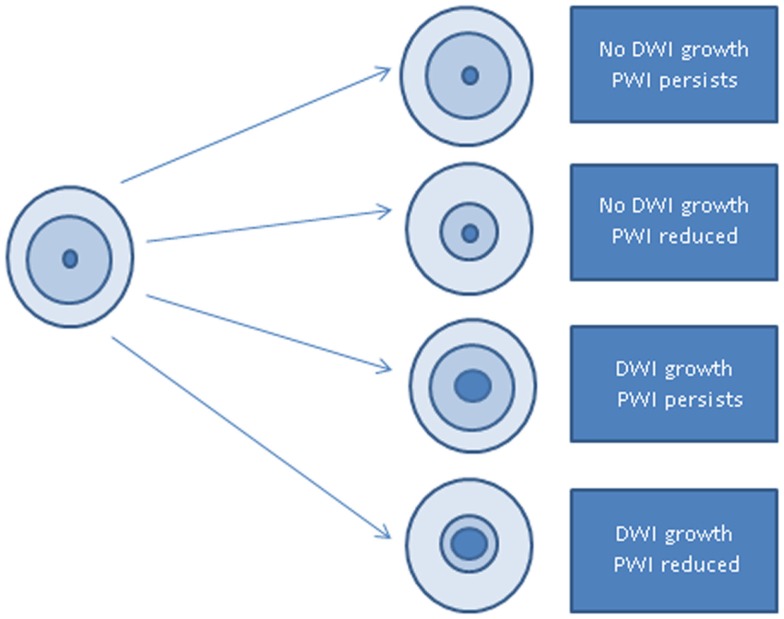
**Schematic of study design: outer circle represents brain; middle circle represents volume of hypoperfusion on PWI; inner circle represents volume of ischemia on DWI**.

We first tested the hypothesis that patients who showed no reperfusion of tissue with 4–5.9 s delay in TTP would show less change in cognitive score than patients who showed reperfusion, irrespective of infarct growth, using a *t*-test in the Stata 13.1 software package^2^ to compare the two groups. Patients were separated into two groups based on whether or not they experienced infarct growth, defined as an increase of 10% in the DWI lesion volume from the first time point to the second time point. These two groups were each further divided based on whether there was reperfusion – defined as a 10% reduction in volume of hypoperfusion on PWI from the first to the second time point (Figure 1). Patients with neither reperfusion nor infarct growth were considered to be the best example of persistent diffusion–perfusion mismatch with PWI threshold of 4–5.9 s delay in TTP. Changes in cognitive scores were compared between the two subgroups (reperfusion vs. persistent hypoperfusion) with *t*-tests.

We also evaluated the Pearson correlation between baseline volume of hypoperfusion (using 4–5.9 s delay in TTP) and (1) final infarct volume measured by volume of ischemia on the second DWI, and (2) persistent hypoperfusion measured by volume of hypoperfusion measured on the second PWI. We also evaluated the Pearson correlation between change in cognitive score and change in volume of hypoperfusion using 4–5.9 s delay in TTP, to test the hypothesis that change in hypoperfusion reflects change in cortical function. However, it is not assumed that there is a direct relationship between the cognitive test score and the change in volume since location of reperfusion likely also plays a significant role. Finally, to determine if the change in volume of hypoperfused tissue contributed to cognitive change independently of change in infarct volume, we carried out a multivariable linear regression analysis, with cognitive change as the dependent variable, and changes in volumes of ischemia on DWI and hypoperfusion on PWI (with thresholds of 4–5.9 and ≥6 s delay) as the independent variables.

## Results

Of the 38 patients identified from the database who met the inclusion criteria, 45% were women; mean age was 61 years. There were 29 patients with a left hemisphere stroke and 9 with a right hemisphere stroke. Table 1 summarizes their etiology and recorded treatment based on retrospective review of their medical records. The mean volume of ischemia on initial DWI was 20.0 cc (range 0–140.4 cc); mean volume of hypoperfusion on initial PWI was 26.8 cc (range 0–111.8 cc) using a threshold of 4–5.9 s TTP, and mean 25.3 (0–116.5 cc) using a threshold of ≥6 s TTP. Mean baseline National Institutes of Health Stroke Scale Score was 6.5 (range 0–24). The mean baseline error rate on the naming test was 57.9% errors (range 0–100% errors). Mean baseline error rate on the cancelation (neglect) test was 43.2% (0–99% errors).

**Table 1:** **Etiology and treatment of patients included**.

	Angioplasty/ embolectomy/ stent/intraarterial tPA/CEA	Blood pressure augmentation with medications^a^	Blood pressure augmentation permissive hypertension +/− intravenous fluids	Anti- platelets + statin only	Anti- coagulation
Intracranial stenoisis/occlusion (*n* = 21)	1	14	6		
Extracranial ICA stenosis (*n* = 2)	2				
Acute ICA occlusion or dissection (*n* = 2)					2
Watershed post CABG (*n* = 1)				1	
Cardioembolic (*n* = 6)					6
Hypercoagulable state (cancer) (*n* = 2)			1		1
Uncertain etiology (OCP, PFO) (*n* = 2)				2	
Small vessel disease (*n* = 2)				2	

*^a^ pressors, midodrine, and/or fludrocortisone*.

*ICA, internal carotid stenosis; CABG, coronary artery bypass graft; OCP, oral contraceptive pills; PFO, patent foramen ovale*.

We first evaluated the change in cognitive test error rate for those who failed to reperfuse (had a persistent diffusion–perfusion mismatch; *n* = 15) compared to those who reperfused (*n* = 23) in the entire group of patients (irrespective of infarct growth). Using a 10% reduction in perfusion abnormality with a threshold of 4–5.9 s delay on TTP maps, there was a significant difference in the change in error rates on cognitive testing between those who failed to reperfuse (mean 3% *increase* in error rate) vs. those who did reperfuse [mean 40% *decrease* in error rate, *t*(36) = 4.1, *p* = 0.0001]. Using a 10% reduction in perfusion abnormality with a threshold of 6 s delay on TTP maps, there was also a significant difference in the change of error rates on cognitive testing, with an increase in errors for those without reperfusion compared to a decrease in error rates for those who did reperfuse [mean 14 vs. −34; *t*(36) = 3.99; *p* = 0.0003].

**Figure 2:**
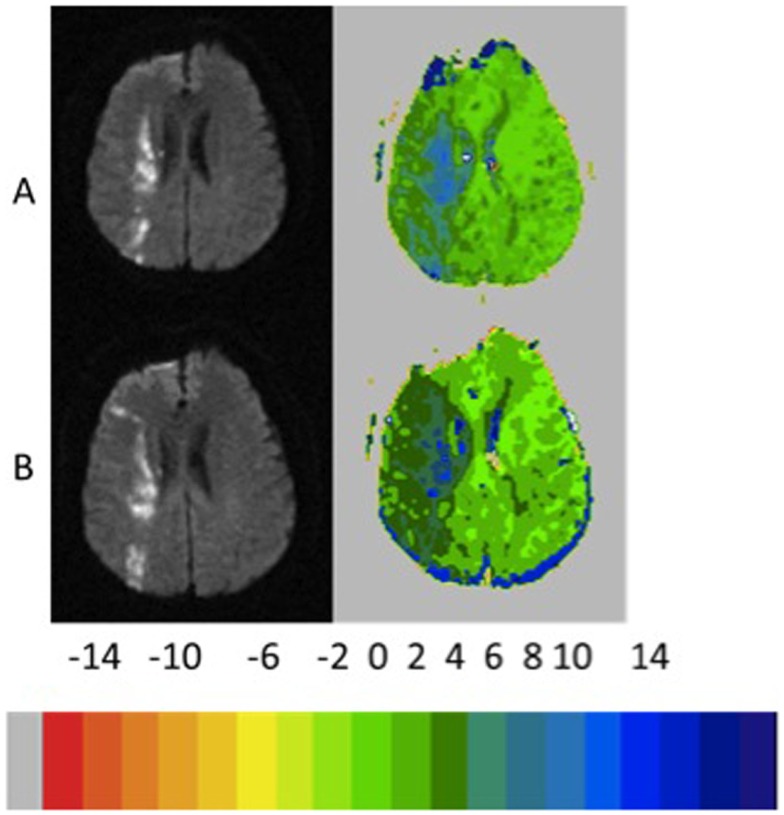
**Example of patient who showed growth of infarct of >10% with <10% reperfusion and persistent severe left neglect on line cancelation task (85 and 71% errors, 3 days later)**. The color key shows the relative delay in TTP (each color corresponds to 2 s delay in TTP arrival of contrast).

**Figure 3:**
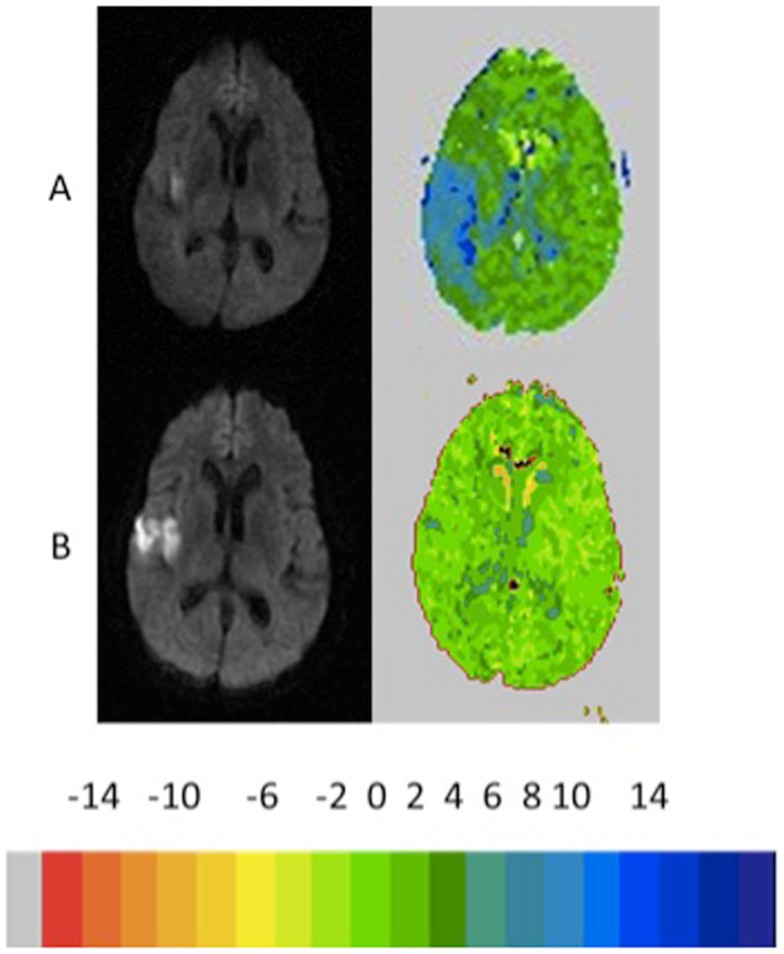
**Example of patient who showed growth of infarct >10% with reperfusion >10% reperfusion with recovery from left neglect on the line cancelation task (99–0% errors, 4 days later)**. The color key shows the relative delay in TTP (each color corresponds to 2 s delay in TTP arrival of contrast).

**Figure 4:**
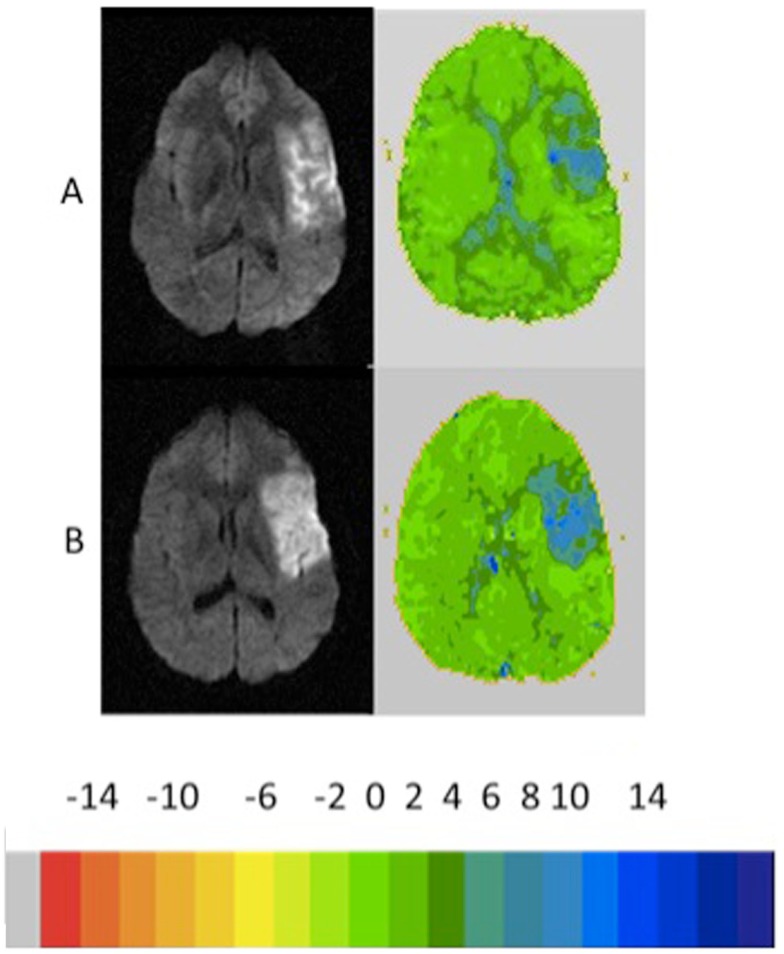
**Example of patient who showed no growth infarct ( <10%) with <10 reperfusion and persistent severe naming impairment (100 and 100% errors, 4 days later)**. The color key shows the relative delay in TTP (each color corresponds to 2 s delay in TTP arrival of contrast).

**Figure 5:**
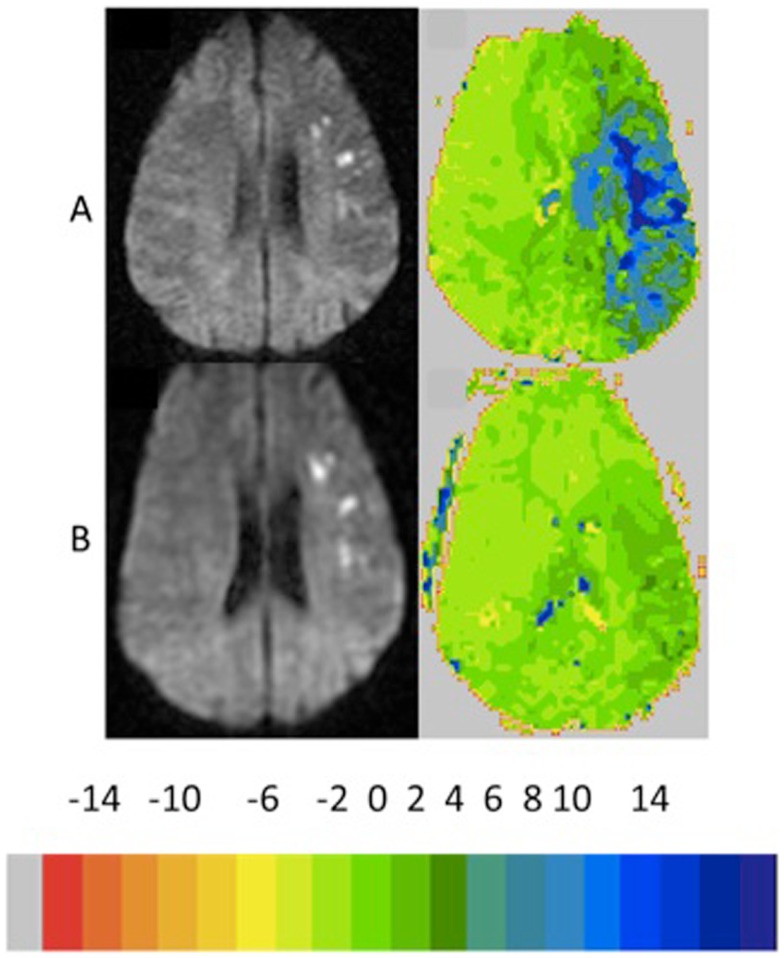
**Example of patient who showed no growth infarct ( <10%) with >10 reperfusion with improvement in naming performance to normal performance (23 and 10% errors, 4 days later)**. The color key shows the relative delay in TTP (each color corresponds to 2 s delay in TTP arrival of contrast).

**Figure 6:**
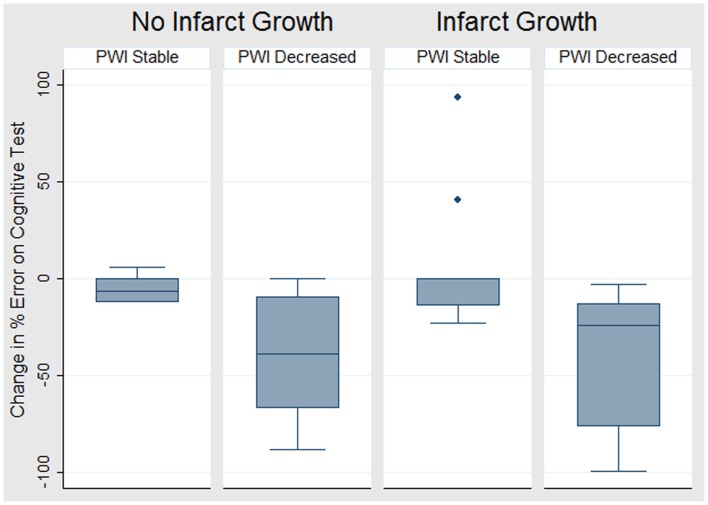
**Boxplot of the change in cognitive score (i.e., change in error rate – negative values indicate improved cognition) for each of four groups defined by change on DWI and PWI**.

Of the 14 patients without infarct growth, the diffusion–perfusion mismatch persisted in 8 (57%) patients who had a mean separation between MRI scans of 3.9 days. Comparing patients with persistent diffusion–perfusion mismatch with those who had reperfused revealed significantly less change in cognitive impairment in those with persistent functional diffusion–perfusion mismatch [−5 vs. −40%; *t*(12) = 2.4; *p* = 0.02]. Examples of patients in each group are shown in Figures 2–5. Figure 6 shows a boxplot of the change in error rates for patients in each of the four groups based on PWI and DWI change. When evaluating the difference between change in cognitive score between those with persistent diffusion–perfusion mismatch when based on a TTP threshold of 6 s instead of 4–5.9 s, the relationship between persistent cognitive deficit and persistent diffusion–perfusion mismatch was not significant but showed the same trend [−2 vs. −34%; *t*(12) = 1.9; *p* = 0.075].

Infarct growth was present in 24 (63%) patients. Even among these patients, many had persistent diffusion–perfusion mismatch (see Figures 3 and 6). Those who failed to reperfuse showed an increase in error rate, while those who reperfused showed a decrease in error rate; the difference was significant, both using a threshold of 4–5.9 s delay in TTP [8 vs. −39%; *t*(22) = 3.3; *p* = 0.002] and using a threshold of 6 s delay in TTP [26 vs. −34%; *t*(12) = 3.6; *p* = 0.002].

There was a *reduction* in DWI lesion volume in eight patients; seven of these patients also showed a >10% reduction in volume of hypoperfusion with 4–5.9 s delay, and associated reduction in errors on cognitive testing. One patient who showed a decrease in DWI lesion showed an *increase* in volume of hypoperfusion on PWI, and an *increase* in error rate on cognitive testing.

**Figure 7:**
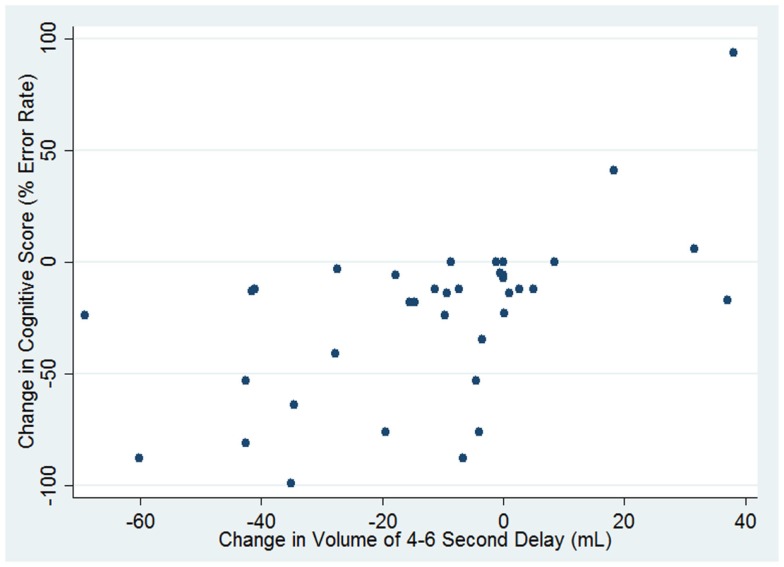
**Correlation between change in cognitive score and change in volume of hypoperfusion (defined as TTP delay of 4–5.9 s)**.

For the patients in this study, the initial volume of hypoperfusion with 4–5.9 s TTP delay did not correlate with the final infarct size (*r* = 0.051; *p* = 0.76). However, the initial volume of hypoperfusion did correlate with the final volume of hypoperfusion (*r* = 0.53; *p* = 0.0006). Moreover, the change in volume of hypoperfusion with 4–5.9 s delay in TTP correlated with the change in cognitive score (*r* = 0.58; *p* = 0.0001) (Figure 7). We merged the scores for naming error rate (left hemisphere stroke) and neglect error rate (right hemisphere stroke) for this analysis, which seemed reasonable, as the scores were comparable in capturing variability: the mean change in naming error rate was −22.8 (±SD 39.3). The mean change in neglect error rate was −22.6 (±SD 30.8).

Finally, to determine if change in volume of hypoperfusion was associated with change in cognitive function independently of change in volume of ischemia on DWI, we carried out a multivariable regression analysis. We found that both change in volume of hypoperfusion with TTP delay of 4–5.9 s (β = 0.542; *p* = 0.002) and change in volume of ischemia on DWI (β = −0.314; *p* = 0.023) independently contributed to change in error rate on cognitive testing (in opposite directions). Together, these two variables accounted for 43% of the variance in change in cognitive score (*r*^2^ = 0.43; *p* = 0.0001). Change in volume of hypoperfusion with ≥6 s delay in TTP was not independently associated with change in cognitive score.

## Discussion

Restoration of blood flow is the goal of all acute ischemic stroke treatments. Preventing infarct and tissue death is a logical target for acute intervention. Previous studies have shown that imaging (PET or MRI) can identify this tissue at risk. However, focusing only on tissue at risk for imminent progression to infarct carries the potential risk of ignoring subacute brain ischemia that might persist beyond the initial event. In this study, we demonstrated that the diffusion–perfusion mismatch with hypoperfusion thresholds of 4–5.9 s delay in TTP can sometimes persist for days and seems to result in persistent cognitive deficits. Those who failed to reperfuse this tissue failed to improve in cognition; while those who reperfused did improve (*p* < 0.001).

In our study, “benign oligemia” defined as tissue with 4–5.9 s delay in TTP was not always benign. Rather, at least some of this tissue seemed to represent “misery perfusion” described by Baron in 1981, which can show functional recovery if reperfused (15). The persistence of perfusion deficits in this range was associated with persistent cognitive deficits when compared with those who reperfused. We confirmed that the initial volume of hypoperfusion with TTP 4–5.9 s delay in TTP did not predict final infarct volume, but did correlate with final volume of hypoperfusion. Furthermore, change in volume of TTP delay correlated with change in cortical dysfunction (measured by change in cognitive score). These results are also consistent with the proposal that tissue with this degree of hypoperfusion (4–5.9 s delay) measured with PWI represents reversibly dysfunctional tissue, even if it is not *always* at imminent risk for progression to infarct. On the other hand, tissue with TTP delay of ≥6 s TTP delay was not associated with change in cognitive function, independently of change in volume of ischemia of DWI and change in volume of hypoperfusion with 4–5.9 s delay. Tissue with ≥6 s TTP delay likely includes much more tissue that is not reversible days after stroke.

There were several limitations to this study. This is a retrospective analysis of prospectively collected data collected over a few years. The MRI scan parameters were not standardized and varied considerably with regard to pulse sequences and magnet strength. The scans at the two time points were not registered, as no high-resolution anatomical scan was obtained with these clinical scans. We were also not able to register the DWI to the PWI on voxel-by-voxel basis; therefore, hypoperfusion defined as 4–5.9 s delay in TTP may have also included some core infarct. However, it is unlikely that the hypoperfused tissue defined with these dual thresholds included much core infarct, because reperfusion of the tissue with 4–5.9 s TTP delay (reduction in that volume) was associated with improvement in cognitive function, independently of change in infarct volume. Cognitive testing may be cumbersome when time is of essence in treatment of acute stroke. However, the simple cognitive tests we used here take <10 min to administer. We also note that these patients were not typical stroke patients. They had a second MRI scan that included PWI 2–10 days after the first. This second scan was generally obtained because persistent hypoperfusion was suspected or to evaluate the effectiveness of intervention to improve perfusion or collateral blood flow (such as temporary induced blood pressure elevation). Thus, there was a relatively small percentage of lacunar strokes, and a relatively large percentage of large vessel stenosis, as the etiology of stroke.

We do not wish to claim that the rate of persistent diffusion–perfusion mismatch observed in this study is representative for an acute stroke population. Rather, our goal was to demonstrate that diffusion–perfusion mismatch with a hypoperfusion threshold of 4–5.9 s delay in TTP can sometimes persist, and appears to represent dysfunctional tissue that can recover function if blood flow is restored. As noted, is not clear how this tissue sometimes survives days after onset – perhaps by misery perfusion from the main arterial supply, or from collateral circulation, either of which may fluctuate with any variable that changes the mean arterial pressure or intracranial pressure (body position, respiratory rate, temperature, and so on). A relatively small number of patients with persistent hypoperfusion of 4–5.9 s delay in TTP showed some infarct growth, indicating that the area of hypoperfusion included some “tissue-at-risk” as well as “benign oligemia.”

Future studies should address some of the limitations of this study, by evaluating both the fate and the clinical consequences of voxels that that are initially non-infarcted (using a specific ADC threshold) but hypoperfused (using various TTP ranges, e.g., 2–3, 3–4, 4–5 s). This analysis should be done on a large, unselected series of acute ischemic stroke patients, using serial imaging registered to high resolution anatomical images, so that DWI and PWI can be registered to each other at each time point and across time points.

Despite its limitations our study indicates that symptomatic, reversible perfusion deficits can be identified by diffusion–perfusion mismatch using a hypoperfusion threshold of 4–5.9 s TTP delay and can occasionally persist for days without progressing to infarction. Thus, the diffusion–perfusion mismatch defined in this way may be clinically useful for identifying patients who might still benefit from reperfusion (e.g., urgent carotid endarterectomy, stenting, efforts to increase collateral blood flow). Failure to restore blood flow to tissue with a TTP delay of 4–5.9 s seems to result in persistent deficits in some cases even if it does not result in imminent progression to infarct. These results do not detract from the usefulness of the diffusion–perfusion mismatch model (or other models of the penumbra) for identifying tissue at risk and patients who require acute intervention, but provide yet another way this imaging might be useful in clinical care.

## Conflict of Interest Statement

The authors declare that the research was conducted in the absence of any commercial or financial relationships that could be construed as a potential conflict of interest.

## Footnotes

^1^http://imagej.nih.gov/

^2^http://www.stata.com/
